# Methylation of the Tumor Suppressor Protein, BRCA1, Influences Its Transcriptional Cofactor Function

**DOI:** 10.1371/journal.pone.0011379

**Published:** 2010-06-29

**Authors:** Irene Guendel, Lawrence Carpio, Caitlin Pedati, Arnold Schwartz, Christine Teal, Fatah Kashanchi, Kylene Kehn-Hall

**Affiliations:** 1 Department of Microbiology, Immunology, and Tropical Medicine, The George Washington University Medical Center, Washington, D. C., United States of America; 2 Department of Pathology, The George Washington University Medical Center, Washington, D. C., United States of America; 3 Breast Care Center, The George Washington University Medical Center, Washington, D. C., United States of America; 4 Department of Molecular and Microbiology, National Center for Biodefense & Infectious Diseases, George Mason University, Manassas, Virginia, United States of America; Bauer Research Foundation, United States of America

## Abstract

**Background:**

Approximately half of hereditary breast cancers have mutations in either BRCA1 or BRCA2. BRCA1 is a multifaceted tumor suppressor protein that has implications in processes such as cell cycle, transcription, DNA damage response and chromatin remodeling. This multifunctional nature of BRCA1 is achieved by exerting its many effects through modulation of transcription. Many cellular events are dictated by covalent modification of proteins, an important mechanism in regulating protein and genome function; of which protein methylation is an important posttranslational modification with activating or repressive effects.

**Methods/Principal Findings:**

Here we demonstrate for the first time that BRCA1 is methylated both in breast cancer cell lines and breast cancer tumor samples at arginine and lysine residues through immunoprecipitation and western blot analysis. Arginine methylation by PRMT1 was observed *in vitro* and the region of BRCA1 504–802 shown to be highly methylated. PRMT1 was detected in complex with BRCA1 504–802 through *in vitro* binding assays and co-immunoprecipitated with BRCA1. Inhibition of methylation resulted in decreased BRCA1 methylation and alteration of BRCA1 binding to promoters *in vivo* as shown through chromatin immunoprecipitation assays. Knockdown of PRMT1 also resulted in increased BRCA1 binding to particular promoters *in vivo*. Finally, following methylation inhibition, Sp1 was found to preferentially associate with hypo-methylated BRCA1 and STAT1 was found to preferentially associate with hyper-methylated BRCA1.

**Conclusions/Significance:**

These results suggest that methylation may influence either the ability of BRCA1 to bind to specific promoters or protein-protein interactions which alters the recruitment of BRCA1 to these promoters. Thus, given the importance of BRCA1 to genomic stability, methylation of BRCA1 may ultimately affect the tumor suppressor ability of BRCA1.

## Introduction

BRCA1 is a tumor suppressor protein that has implications in processes such as cell cycle, transcription, DNA damage response, and chromatin remodeling. One way to explain the multifaceted nature of BRCA1 is that it exerts its many effects through modulating transcription of various factors. BRCA1 was first implicated in transcription when the C-terminus (amino acid, aa, 1560–1863) fused to Gal4 was able to activate transcription [1], with aa 1760–1863 being the minimal transactivation domain (TAD). Within this TAD are two BRCA1 C-terminus (BRCT) motifs that are found in a large family of proteins important for DNA damage response, such as DNA ligase IV, p53BP1, and base excision response scaffold protein XRCC1 [2]. Since that time, numerous other findings have served to strengthen the connection between transcription and BRCA1. For instance, BRCA1 is part of the RNA polymerase II holoenzyme complex [3], [4], [5]. BRCA1 also interacts with multiple cofactors and transcription factors, including CBP/p300, Sp1, STAT1, Estrogen Receptor and BRG1 [6], [7]. Among the genes found to be transactivated by BRCA1 are MDM2, BAX, p21/WAF1, p27/KIP1, and GADD45α with p21/WAF1 and GADD45α transactivation being independent of p53 [8], [9], [10], [11], [12], [13].

Proteins involved in transcription are often regulated through posttranslational modifications (PTMs), such as acetylation and methylation. Acetylation is broadly linked with activation, whereas methylation can have either activating or repressing effects depending on the amino acid that is modified and the nature of the modification (mono-, di-, or tri-methylation). One well known examples of this is the transcription factor p53, where K372 monomethylation and K370 dimethylation result in stabilization and activation of p53 versus K370 and K382 monomethylation, which results in transcriptional repression [14]. To date, the known PTMs of BRCA1 include phosphorylation and ubiquitinylation [7]. The phospho-BRCA1 predominates in S phase and subsequently becomes dephosphorylated after M phase [7]. This cell cycle dependent phosphorylation occurs in the absence of DNA damage and is accomplished by multiple protein kinases including cyclin A/cdk2, cyclin E/cdk2, cyclin D/cdk4, and aurora-A [7]. In addition, BRCA1 is phosphorylated following DNA damage by ATR and ATM kinases [7]. Although epigenetic changes to the BRCA1 gene have been extensively researched at the DNA-level, proteome research has focused on phosphorylation of BRCA1 by DNA damage protein kinases, while little information is known about the effects of other PTM events.

Protein methylation can occur on both lysine and arginine residues. Arginine methylation is carried out by a family of protein arginine methyltransferases (PRMT), which contains eleven family members to date [15]. These enzymes utilize S-adenosyl methionine (AdoMet) as a methyl donor [16], and can be further subdivided into type I and type II enzymes. Type I enzymes form monomethylarginine (MMA) and asymmetric dimethylarginine (aDMA) and type II enzymes catalyze the formation of MMA and symmetric dimethylarginine (sDMA) [17]. PRMT1, PRMT2, PRMT3, PRMT4/CARM1, PRMT6, and PRMT8 are type I enzymes; while PRMT5, PRMT7, and PRMT9 are type II enzymes. PRMT1 is the most predominant methyltransferase in mammalian cells [18] and is responsible for the majority of arginine methylation, thus the vast majority of research has been focused on this enzyme [19]. A number of PRMT1 substrates have been identified including 53BP1, histone H4, MRE11, nucleolin, RNA helicase A, SAM68, and ERα [20], [21], [22], [23]. Arginine methylation can regulate multiple cellular processes including transcription [24], [25], [26], [27], [28], [29], protein-protein interactions [30], nuclear trafficking [31], [32], transcriptional elongation, DNA damage response [22], and cell cycle checkpoints [22]. Lysine methylation occurs mainly through the SET domain family of proteins. These enzymes were originally termed histone methyltransferases due to their ability to methylation various histone protein residues [33], but in light of the identification of many non-histone protein substrates they are now referred to as protein lysine methyltransferases (PKMTs). The exception to this is the DOT1 family of lysine methyltransferases. Lysine residues can be mono-, di-, or tri-methylated [34], [35].

Here we have identified for the first time the arginine and lysine methylation of BRCA1 in tissue culture cell lines as well as breast tumor tissue samples. We show that BRCA1 is methylated by PRMT1 *in vitro* within the 504–802 region. PRMT1 was detected in complex with BRCA1 504–802 through *in vitro* binding assays and co-immunoprecipitated with BRCA1. Inhibition of methylation resulted in decreased BRCA1 methylation and alteration of BRCA1 binding to promoters *in vivo*. Knockdown of PRMT1 also resulted in increased BRCA1 binding to particular promoters *in vivo*. Finally, following methylation inhibition, Sp1 was found to preferentially associate with hypo-methylated BRCA1 and STAT1 was found to preferentially associate with hyper-methylated BRCA1. These findings indicate that BRCA1 is posttranslationally modified through methylation by PRMT1 and that this methylation influences its ability to bind to different promoters *in vivo*.

## Results

### BRCA1 is methylated in breast cancer cell lines

**Figure 1:**
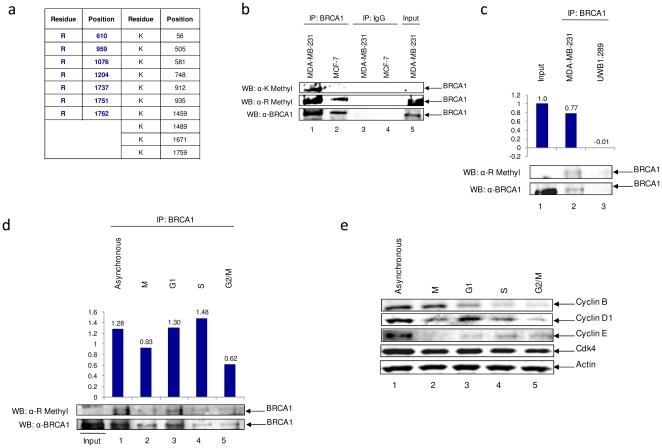
BRCA1 is methylated at both arginine and lysine residues in breast cancer cell lines. (**a**) Predicted BRCA1 methylation sites generated by Memo: Methylation Modification Prediction Server (http://www.bioinfo.tsinghua.edu.cn/~tigerchen/memo.html) R = Arginine and K = Lysine. (**b**) Two milligram of whole cell protein extracts from MCF-7 and MDA-MB-231 cells were immunoprecipitated with either BRCA1(C-20) or rabbit normal IgG antibody, separated on a 4–20% gel by SDS-PAGE, and western blotted with antibodies against K-methyl, R-methyl and BRCA1(C-20). Input represents 1/10 of immunoprecipitated material. Results are representative of three independent experiments. (**c**) One milligram of whole cell protein extracts from MDA-MB-231 and BRCA1-null UWB1.289 cells were immunoprecipitated with either BRCA1(C-20) or rabbit normal IgG antibody, separated on a 4–20% gel by SDS-PAGE, and western blotted with antibodies against R-methyl and BRCA1(C-20) in order to validate observed methylated bands. Densitometry of R-methylation was normalized to amount of detected immunoprecpipitated BRCA1, background subtracted based on IgG counts. (**d**) One milligram of whole cell protein extracts from synchronized MDA-MB-231 cells were immunoprecipitated with either BRCA1(C-20) or rabbit normal IgG antibody, separated on a 4–20% gel by SDS-PAGE, and western blotted with antibodies against R-methyl and BRCA1(C-20). Densitometry of R-methylation was normalized to amount of detected immunoprecpipitated BRCA1, background subtracted based on IgG counts. Input represents 1/10 of immunoprecipitated material. (**e**) Whole cell protein extracts from synchronized MDA-MB-231 cells were separated on a 4–20% gel by SDS-PAGE, and western blotted with antibodies against Cyclin B(H-433), Cyclin D1(M-20), Cyclin E(C-19), cdk4(H-303) and actin(C-11).

Methylation of proteins can have either activating or repressive effects depending on the amino acid that is modified (arginine-R or lysine-K) and whether it is mono-, di-, or tri-methylated [36]. For example, K4 methylation on histone H3 is an indicator of actively transcribed genes [36]. In contrast, methylation of histone H3K9, H3K27, and H4K20 is involved in heterochromatin formation and gene silencing [36]. To date there are no reports of BRCA1 protein methylation. Based on the role of BRCA1 in transcription and the influence of methylation on various transcription factors, we speculated that BRCA1 may be methylated. *In silico* analysis revealed a total of seven R and ten K residues in BRCA1 that could potentially be methylated (Figure 1a). Interestingly, two of these residues, R1076 and R1751 have known BRCA1 mutations, R1076T, R1751Q and R1751P, according to the Breast Cancer Information Core (http://research.nhgri.nih.gov/bic/). To determine if methylated BRCA1 could be detected in breast cancer cell lines, two cell lines, MCF-7 and MDA-MB-231, were analyzed. BRCA1 was immunoprecipitated and western blot analysis performed with anti-K methyl, anti-R methyl and anti-BRCA1 antibodies. Results in Figure 1b indicated that BRCA1 is methylated on both K and R residues in MDA-MB-231 cells, but only R methylation could be detected in MCF-7 cells. These cell lines have very distinct characteristics, with MDA-MB-231 being triple negative and MCF-7 being ER and PR positive. In addition, MDA-MB-231 is a highly metastatic cell line, whereas MCF-7 is not. To demonstrate that the methyl band observed is specific to BRCA1, BRCA1 was immunoprecipitated from MDA-MB-231 and UWB1.289 BRCA1 negative cells (Figure 1c). No methyl band was detected in UWB1.289 cells, indicating that the methylated band is specific to BRCA1. To further characterize BRCA1 methylation status, MDA-MB-231 cells were synchronized by nocodazole treatment and cell populations collected at various stages of the cell cycle. Results in Figure 1d indicate that R methylation can be detected throughout the cell cycle with no drastic changes, suggesting that this PTM may not be cell cycle regulated. Synchronization was verified by western blotting for cyclin B, cyclin D1, cyclin E (Figure 1e), with cdk4 and actin serving as controls. Collectively, these results indicate that BRCA1 is methylated in breast cancer cell lines. This is the first time to our knowledge that BRCA1 has been shown to be methylated.

### BRCA1 is methylated in *ex vivo* patient samples

**Figure 2:**
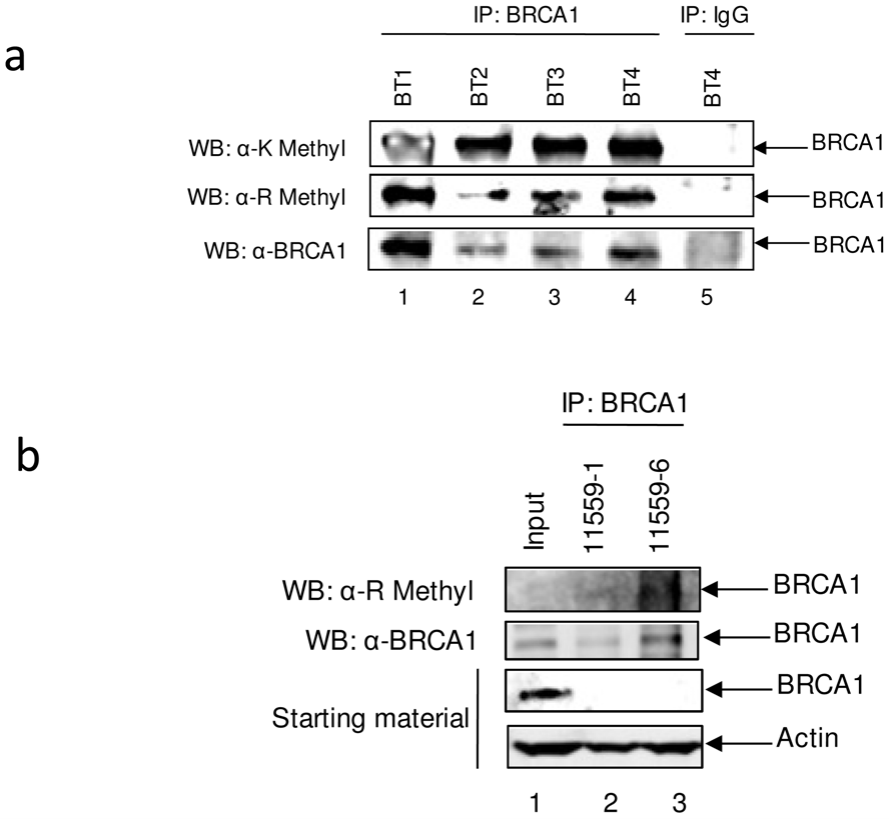
Methylation of BRCA1 in *ex vivo* breast tumor samples. (**a**) Solid breast tumor tissue was ground with a mortar and pestle in the presence of liquid nitrogen to create a powdered tissue. RIPA buffer was added to the powdered tissue, the sample vortexed for 60 seconds, and placed on ice for 45 minutes. Samples were homogenized with a syringe and needle, followed by centrifugation at 14,000 g for 10 minutes. Two milligram of whole cell protein extracts from four different breast tumor samples (BT1-4) were immunoprecipitated with either BRCA1(C-20) or rabbit normal IgG antibody, separated on a 4–20% gel by SDS-PAGE, and western blotted with an anti-K methyl, anti-R methyl and anti-BRCA1 antibodies. Results are representative of two independent experiments. Characteristics of tumor tissues are as follows: BT1: infiltrating ductal carcinoma, age 55, African American, ER negative, PR negative, HER2 negative, p53 negative. BT2: infiltrating lobular carcinoma, age 83, Caucasian, ER positive, PR negative, HER2 negative, p53 negative. BT3: variant papillary serous type of ductal carcinoma, age 54, African American, ER negative, PR negative, HER2 negative, p53 negative. BT4: infiltrating ductal carcinoma, age 68, Caucasian, ER negative, PR negative, HER2 negative, p53 positive. (**b**) Matched breast tumor tissue was processed as described. Four milligram of whole cell protein extract were immunoprecipitated with either BRCA1(C-20) or rabbit normal IgG antibody, separated on a 4–20% gel by SDS-PAGE, and western blotted with an anti-R methyl and anti-BRCA1 antibodies. Input represents 1/5 of immunoprecipitated material.

To determine if BRCA1 is also methylated in *ex vivo* patient samples, four different breast tumor tissue samples (BT1-4) were tested. Interestingly, 3 of the 4 samples were triple negative breast cancers (BT1, 3, and 4), which are extremely difficult to treat. BT3 was ER positive, PR negative and does not have HER-2 overexpression. We immunoprecipitated BRCA1 from BT1-4 and western blotted with anti-K methyl, anti-R methyl and anti-BRCA1 antibodies (Figure 2a). Our results indicate that BRCA1 is methylated at both K and R residues (lanes 1–4) in all four breast tumor patient samples, while no methylation was observed with the negative control IgG IP (lane 5). To investigate methylation status in normal tissue, a matched tumor sample was equally processed and immunoblotted with anti-R methyl antibody. BRCA1 appears to be expressed and methylated in higher amounts in the one normal tissue examined (Figure 2b, compare lanes 2 and 3). Of interest, BRCA1 levels were not detected when western blotting the non-immunoprecipiated material, indicating that these tissues samples express very low levels of BRCA1. Tumor samples 11559-1 is an ER positive, PR negative, non-overexpressed HER-2 invasive ductal carcinoma, similar to BT2 in panel 2a. Larger sample sizes are necessary to determine if BRCA1 methylation occurs more frequently in particular types of breast cancer or in normal breast tissue. These preliminary results demonstrate that both K- and R-methylation are detected in normal and breast tumor tissue samples.

### PRMT1 methylates and associates with BRCA1

**Figure 3:**
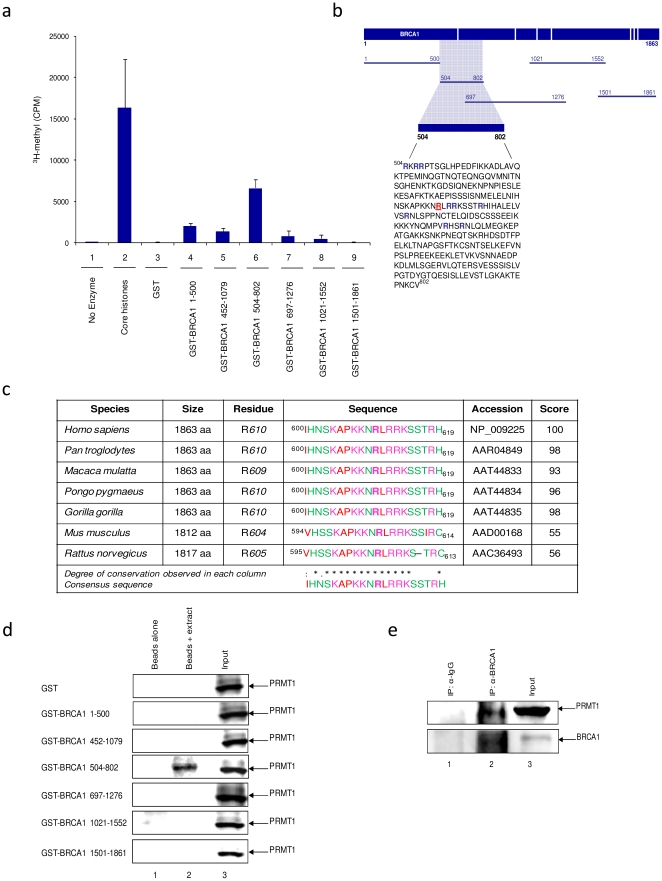
PRMT1 methylates and associates with BRCA1. (**a**) GST-BRCA1 constructs (0.5 µg) and core histones (1 µg) were incubated with purified recombinant PRMT1 enzyme (0.2 µg) in the presence of 0.55 µCi *S*Adenosyl- L-[*methyl*-3H] methionine. GST was used as negative control and all GST-BRCA1 construct methylation levels normalized to background GST methylation levels. An average result of two independent experiments run in triplicates is shown. (**b**) The amino acid sequence of BRCA1 504–802 with arginine (R) residues within the 540–696 minimal region highlighted in blue and bolded, and the predicted methylated arginine residue highlighted in red, underlined and bolded. (**c**) Multiple sequence alignment generated by Clustal W of four non-human primates and two rodent BRCA1 full length sequences. Score represents pairwise alignment against full length human BRCA1. Amino acid residues are color-coded according to physical properties: basic (magenta), small hydrophobic (red) and hydroxyl + amine + basic (green). Consensus symbols for degree of conservation observed is represented by “*” (residues in column are identical in all seven sequences), “:” (conserved substitutions observed), and “.” (semi-conserved substitutions observed). (**d**) Two milligram of HeLa whole cell protein extract was incubated with 0.5 µg GST-BRCA1 constructs, beads were washed twice with TNE_150_ + 0.1% NP-40 and once with TNE_50_ + 0.1% NP-40, separated on a 4–20% gel by SDS-PAGE, and probed with an antibody against PRMT1. Input represents 1/10 of immunoprecipitated material. (**e**) Two milligram of HeLa whole cell protein extract were immunoprecipitated with anti-BRCA1 and anti-IgG antibodies, beads were washed twice with TNE_150_ + 0.1% NP-40 and once with TNE_50_ + 0.1% NP-40, separated on a 4–20% by SDS-PAGE, and probed with an anti-PRMT1 antibody. Input represents 1/10 of immunoprecipitated material. Results are representative of two independent experiments.

Results above indicate that BRCA1 protein is methylated in both cancer cell lines and patient samples. We were interested in the site of BRCA1 arginine methylation and for that purpose, GST-BRCA1 constructs spanning BRCA1 protein were used for *in vitro* methyltransferase assays. Due to the lack of a previously identified enzyme, GST-PRMT1 was chosen for this analysis because PRMT1 is responsible for approximately 85% of all arginine methylation [19]. GST-BRCA1 constructs spanning the entire protein (Figure 3a) were incubated in a reaction mixture containing GST-PRMT1 enzyme, buffer, and *S*-Adenosyl-L [*methyl*-3H] methionine as a source of radio-labeled methyl groups. Core histones were used as a positive control, as PRMT1 has been shown to methylate histone H4 [17], [37] and GST was used as negative control. BRCA1 fragment 504–802 was consistently the most highly methylated in repeated experiments, while no methylation was detected for GST-BRCA1 1501–1861(Figure 3a). Lower levels of methylation were observed with BRCA1 1–500, 452–1079, and 1021–1552. As BRCA1 504–802 overlaps with GST-BRCA1 697–1276, which exhibited no methylation, amino acids 504–696 are the minimal area necessary for the observed methylation. According to the predicted methylation sites in Figure 3a, the only arginine predicted to be methylated within the 504–802 region is R610 (highlighted in red in Figure 3b). R610 is not the typical GAR motif present in many PRMT1 substrates, but is an RXR sequence, which is methylated in Poly(A)-binding protein II (PABPII) by PRMT1 [38]. Therefore, it is possible that other arginine residues within BRCA1 504–802 are methylated (highlighted in blue in Figure 3b).

As BRCA1 is a poorly conserved protein, we were interested to determine if the predicted methylation site was a conserved residue. Full length BRCA1 sequences from human, four non-human primates and two rodents were aligned using Clustal W in order to analyze conservation of the predicted site within our identified minimal methylation region. Of interest, MeMo-predicted methylation site and flanking residues of human BRCA1 ^600^IHNSKAPKKNRLRRKSSTRH^619^ are highly conserved in all sequences analyzed, even in rodent BRCA1 sequences which presented approximately 55 percent identity to human BRCA1 (Figure 3c).

To confirm an interaction between PRMT1 and BRCA1, all the GST-BRCA1 constructs utilized for the methyltransferase assay were used for a pull-down assay with total cell lysates from HeLa cells. HeLa cells were chosen as they display high levels of PRMT1 expression in contrast to other breast cancer cell lines (data not shown). An anti-PRMT1 western blot of the GST pull-down revealed that the only detectable interaction between PRMT1 and BRCA1 was occurring at the previously identified region of 504–802 (Figure 3d). To further corroborate this interaction, whole cell extracts were prepared and immunoprecipitated with BRCA1 or IgG and western blotted with anti-PRMT1. BRCA1-PRMT1 interaction was observed specifically with the BRCA1 immunoprecipitation and not with the IgG (Figure 3e). Collectively, these results indicate that PRMT1 methylates BRCA1 *in vitro*, that the region of 504–802 is methylated, and that physical interaction of PRMT1-BRCA1 is only detectable at the 504–802 region of BRCA1.

### Methylation of BRCA1 alters promoter binding *in vivo*

**Figure 4:**
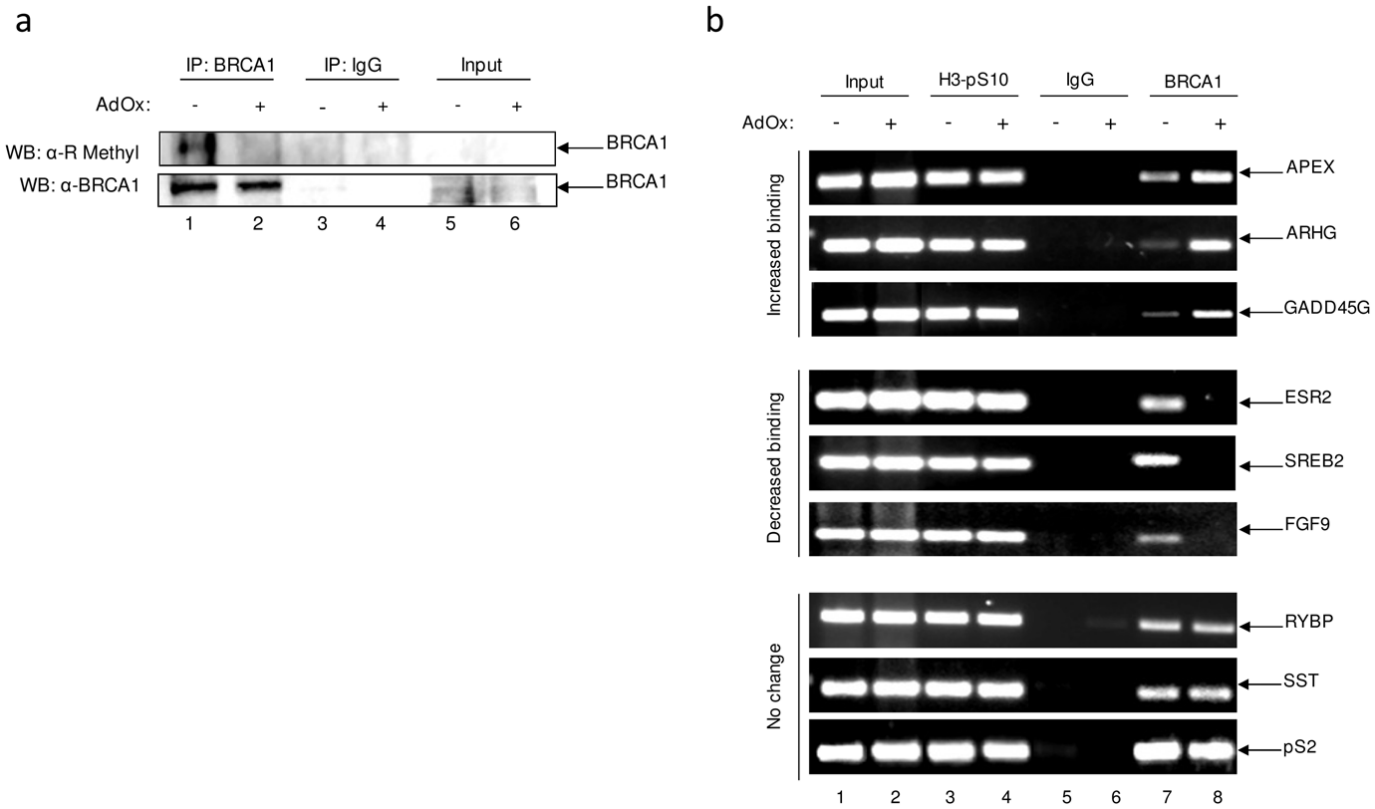
Methylation status of BRCA1 alters BRCA1-DNA interactions at specific promoters *in vivo.* (**a**) MDA-MB-231 cells were treated with AdOx (30 µM) in order to observe BRCA1 methylation inhibition upon treatment. Two milligram of MDA-MB-231 whole cell protein extract was immunoprecipitated with anti-BRCA1 and anti-IgG antibodies, separated on a 4–20% gel by SDS-PAGE, and probed with anti-K methyl antibody. Blot was stripped and reprobed with anti-BRCA1 antibody. Input represents 1/10 of immunoprecipitated material. Results are representative of two independent experiments. (**b**) MDA-MB-231 cells were treated with AdOx (30 µM) for 48 hours prior to being collected for ChIP analysis. Antibodies used for ChIP were anti-BRCA1 (10 µg), anti-IgG (10 µg), and anti-histone H3-phosphorylated at S10 (H3-pS10, 5 µg). PCR products were run on a 2% agarose gel and visualized with ethidium bromide staining. Results are representative of two independent experiments.

The internal region of BRCA1 (aa 452–1079) contains two DNA binding domains, DB1 at aa 498–663 and DB2 at aa 936–1057 [39]. BRCA1 binds to branched DNA structures [40] as well as the TTC(G/T)GTTG consensus sequence [41]. Therefore, it can influence DNA damage repair pathways such as homologous recombination as well as being a transcriptional cofactor. We have previously shown that BRCA1 can bind to eight novel promoters containing the TTC(G/T)GTTG consensus site [42] and wondered whether methylation of BRCA1 influences the binding of BRCA1 to these promoters. To this end, MDA-MB-231 cells were treated with the methyltransferase inhibitor adenosine dialdehyde (AdOx) [43]. AdOx treatment resulted in decreased BRCA1 arginine methylation (Figure 4a), further verifying that BRCA1 is methylated and providing a system to test the influence of methylation on the function of BRCA1. ChIP assays from untreated and AdOx treated cells were performed using antibodies against histone H3-pS10 (positive control), IgG (negative control) and BRCA1. Interestingly, BRCA1 binding was increased at the AP endonuclease (APEX), Ras homolog gene family member G (ARHG) and the growth arrest and DNA damage inducible family member G (GADD45G) when BRCA1 is hypomethylated (Figure 4b). Conversely, estrogen receptor beta (ESR2), G-protein coupled receptor 85 (SREB2) and the mesothelial fibroblast growth factor 9 (FGF9) exhibited a marked decrease of BRCA1 binding upon protein methylation inhibition. Lastly, the RING1 and YY1 binding protein (RYBP) promoter, somatostatin (SST) and the estrogen regulated gene, pS2 displayed no change in promoter binding between BRCA1 methylation states (Figure 4b). These results indicate that methylation of BRCA1 could play an important role in BRCA1 DNA binding at specific promoters *in vivo*.

### Decreased levels of PRMT1 alters BRCA1 promoter binding *in vivo*

**Figure 5:**
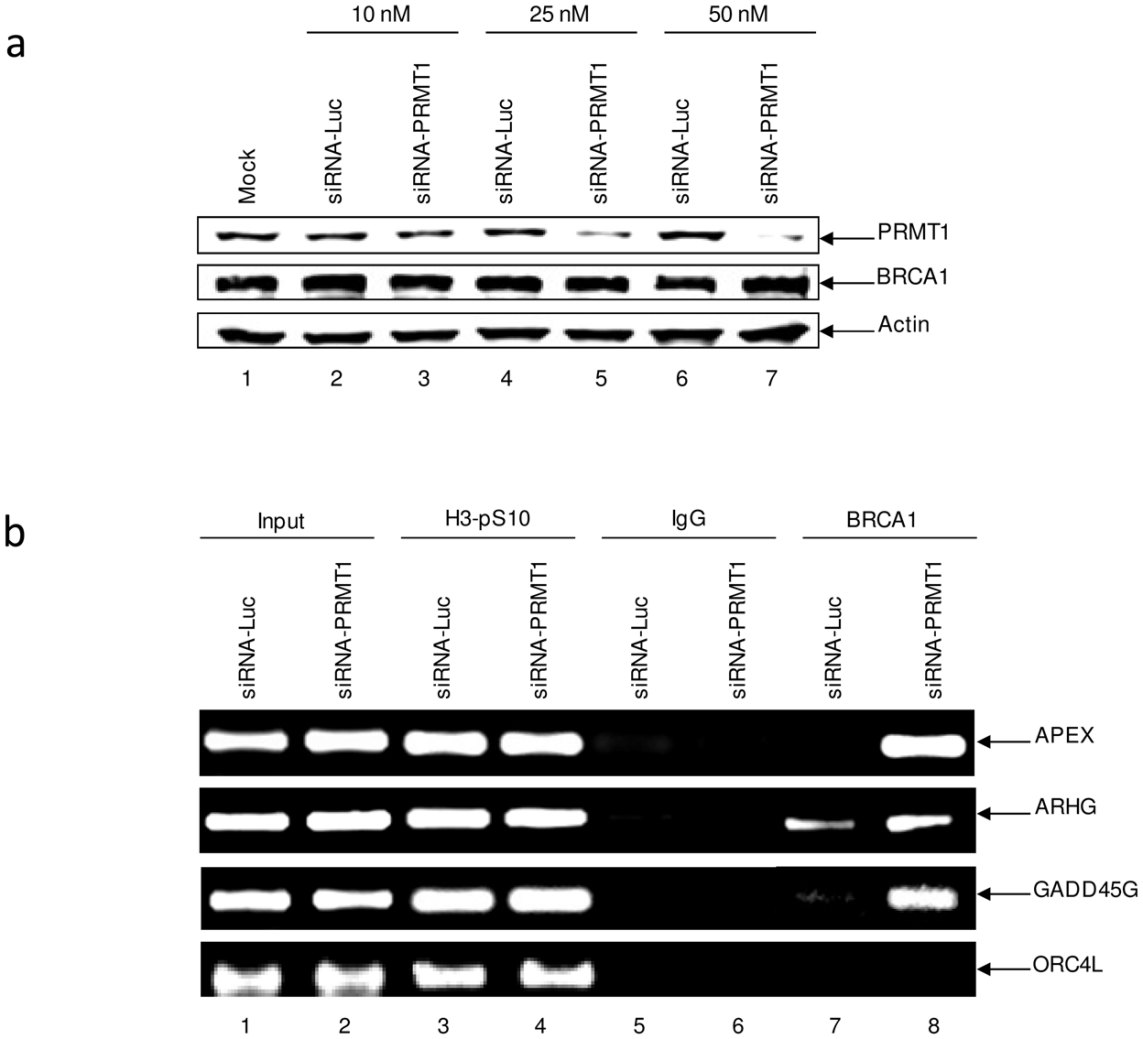
Decreased levels of PRMT1 alters BRCA1 promoter binding *in vivo.* (**a**) HeLa cells were transfected with different concentrations of PRMT1 siRNA (10, 25, 50 nM) following manufacturer's instructions. Results are representative of two independent experiments. (**b**) HeLa cells transfected with 50 nM Luc or PRMT1 siRNA were collected for ChIP analysis. Anti-BRCA1 (10 µg), anti-IgG (10 µg), and anti-histone H3-phosphorylated at S10 (H3-pS10, 5 µg) antibodies were used for ChIP analysis. PCR products were run on a 2% agarose gel and visualized with ethidium bromide staining. Results are representative of two independent experiments.

To further characterize the specific impact of BRCA1 methylation by PRMT1, PRMT1 cellular levels were decreased by means of RNAi. A titration of PRMT1 siRNA indicated that at 50 nM, PRMT1 protein levels were decreased by 90% (Figure 5a). Therefore, 50 nM PRMT1 siRNA was utilized for ChIP assays to determine the influence of PRMT1 on BRCA1 DNA binding. Consistent with ChIP results from the AdOx treated cells, PRMT1 knockdown resulted in a dramatic increase of BRCA1 DNA binding to the APEX and GADD45G promoters (Figure 5b, lanes 7–8). A modest increase in binding was also observed at the ARHG gene. ORC4L was used as an internal control for no known interaction with BRCA1 [42]. No change in H3-pS10 was observed following PRMT1 knockdown. These results further suggest that PRMT1 methylates BRCA1 and that its capacity to methylate affects binding of BRCA1 to its responsive promoters.

### Methylation of BRCA1 alters protein-protein interactions

**Figure 6:**
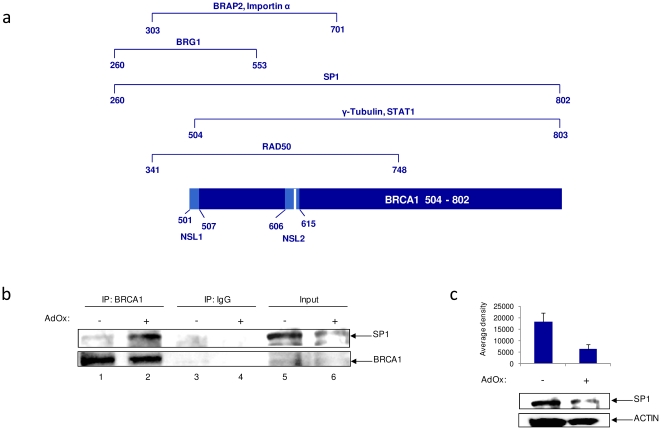
BRCA1 methylation status alters protein-protein interactions at the 504-802 region. (**a**) Schematic of BRCA1 504-802 primary sequence depicting important protein-protein interactions and domains that could be affected by the methylation of this region. (**b**) MDA-MB-231 cells were treated with AdOx (30 µM) in order to observe BRCA1 methylation inhibition upon treatment. Two milligram of MDA-MB-231 whole cell protein extract was immunoprecipitated with anti-BRCA1 or anti-IgG antibodies, separated on a 4-20% gel by SDS-PAGE, and western blotted using antibodies against Sp1 and BRCA1 proteins. Input represents 1/10 of immunoprecipitated material. Results are representative of two independent experiments. (**c**) MDA-MB-231 cells were treated with AdOx (30 µM) and whole cell extract separated on a 4-20% gel by SDS-PAGE, and probed with anti-Sp1 antibody. Densitometry was averaged from three independent immunoblots.

BRCA1 is known to participate in many protein-protein interactions. A schematic diagram of the BRCA1 504–802 protein region is displayed in Figure 6a to indicate protein-protein interactions that have been reported within the region and thus interactions that may be affected by the methylation status of BRCA1. These include transcriptional coactivator BRG1 [44], DNA repair protein RAD50 [45], regulators of nuclear BRCA1 transport importin-α and BRAP2 [46], [47], centrosome and microtubule component γ-tubulin [48], and transcription factors Sp1 and STAT1 [49], [50]. To investigate whether hyper- or hypo-methylation status of BRCA1 interfered with protein-protein interactions, AdOx treated MDA-MB-231 whole cell protein extracts were immunoprecipitated with anti-BRCA1 or anti-IgG antibodies. Immunoblotting of electrophoresed proteins revealed Sp1 preferentially bound to hypo-methylated BRCA1 (Figure 6b, compare lanes 1 and 2). To determine if AdOx has an effect in the abundance of Sp1 (as observed in Figure 6b, lanes 5 and 6), MDA-MB-231 cells were subjected to AdOx treatment and immunoblotted with anti-Sp1 (Figure 6c). Densitometry from three independent experiments indicates that Sp1 total levels tend to decrease in AdOx treated cells, highlighting the relevance of observed increase in BRCA1-Sp1 interaction in Figure 6b lanes 1 and 2. In contrast, we observed STAT1 preferentially associated with hyper-methylated BRCA1 (data not shown). These results suggest that methylation of BRCA1 affects protein-protein interactions.

## Discussion

The molecular function of BRCA1 has been subject of focused studies since it was cloned in 1994 [51]. Extensive studies have characterized BRCA1 as a multifaceted tumor suppressor protein due to its role in cell cycle progression, DNA repair and DNA damage response processes, transcriptional pathway regulation and apoptosis [7]. BRCA1 is regulated through phosphorylation by the DNA damage response kinases, hCds1/Chk2, ATM, and ATR, following DNA damage produced by ionizing radiation, UV, or DNA damaging inducing chemicals such as mitomycin C [52], [53], [54], [55], [56]. Our *in vitro* and *in vivo* analyses indicate another avenue for BRCA1 regulation through arginine methylation, and PRMT1 as a cellular arginine methyltransferase candidate for this methylation. Interestingly, methylation of BRCA1 by PRMT1 indicates a regulatory mechanism for BRCA1 binding to particular promoters as well as protein-protein interactions.

We have demonstrated that BRCA1 is methylated both in breast cancer cell lines and breast cancer tumor samples. Both arginine and lysine methylation was detected. Interestingly, lysine methylation was only detected in MDA-MB-231 cells but not MCF-7, while arginine methylation was detected in both. Both cell lines were obtained from pleural effusions, but differ in their characteristics. *In vitro*, MDA-MB-231 cells display a highly invasive phenotype in contrast to MCF-7 cells, while they both have the ability to form *in vivo* tumors in mice [57], [58]. In correlation with breast cancer, MDA-MB-231 cells are triple negative and posses a mutant p53. MCF-7 cells are double positive, negative for HER2 and posses wild-type p53 [58], [59], [60]. It is tempting to speculate that methylation patterns for both lysine and arginine may be linked to phenotypical characterization of breast cancer types. However, a much larger sample size is needed to draw a clear conclusion in this regard. Arginine methylation by PRMT1 was observed *in vitro* and the region of BRCA1 504–802 was highly methylated. One well known PRMT consensus methylation sequence is the arginine and glycine-rich (GAR) motif (i.e. repeating RGG sequences), which is recognized by PRMT1, 3, 5, 6, and 8 [61]. However, more recently a focused peptide library screen was used to identify additional sequences methylated by PRMT1 [62]. The authors demonstrated that additional sequences such as “RLG”, “RYG”, “RFG”, “RTG”, and “RKG” were substrates for PRMT1. In addition, other PRMTs, such as PRMT4 have no known consensus site, which hinders the identity of arginine methylated proteins. The predicted methylation site at residue 610 harbors a “RXR” sequence, where X is occupied by a leucine, making it the most likely candidate for methylation within that area.

Upon methylation inhibition, *in vivo* BRCA1 binding to the APEX, ARHG and GADD45G promoters was increased. BRCA1 binding to the ESR2, SREB and FGF9 gene promoters was hindered. In addition, BRCA1 binding to RYBP, SST and pS2 gene promoters was unaffected. These results suggest that methylation may influence either the ability of BRCA1 to bind to specific promoter or protein-protein interactions which alters the recruitment of BRCA1 to these promoters. As can be observed in Figure 4a, AdOx treatment abolished detectable levels of arginine methylation in BRCA1. AdOx inhibits activity of all cellular methyltransferases, thus its effect regarding PRMT1 is non-specific. However, BRCA1-DNA interaction at the APEX, ARHG and GADD45G promoters upon siRNA-mediated knockdown of PRMT1 mimicked the results observed when cells were treated with the methyltransferase inhibitor. The difference between the levels of increased binding observed in AdOx-treated versus PRMT1 knockout samples may be due to compensatory mechanisms by other PRMTs or lysine methylation. Preliminary studies demonstrated that SETDB1, a PKMT member of the SUV39 family of SET-domain containing proteins, methylated BRCA1 *in vitro* (data not shown). Neither the site of methylation nor the influence of lysine methylation on BRCA1 is known, but will be the focus of future studies.

Protein-protein interactions at the BRCA1 504–802 region involve several proteins that are important for transcription pathways and in particular, protein localization. These include BRAP2, importin-α, BRG1, Sp1, STAT1 and γ-tubulin. BRCA1 localization also plays an important role in protein function, with both cytoplasmic and nuclear targets. Subsequently, BRCA1 has been found to posses two nuclear localizations signals (NLS) as well as one nuclear export sequence (NES) that guide the shuttling process of BRCA1 [63], [64], [65]. Albeit, mechanisms for this shuttling process are not clearly understood. Interestingly, both NLS are located in the vicinity of the identified BRCA1 region that is being methylated. Specifically, NSL1 is located at residues 501–507 and NSL2 at residues 606–615. Furthermore, phosphorylation of T508 at the Akt concensus phosphorylation motif immediately adjacent NSL1 resulted in cytoplasmic accumulation of BRCA1 [66], suggesting that methylation of this region may possess similar shuttling regulatory mechanisms. PRMT1 is also regulated through nucleo-cytoplasmic shuttling [67], [68]. Importantly, enzymatic activity is required for this shuttling process where a catalytically inactive mutant of PRMT1, rapidly accumulates in the nucleus. The nuclear export of PRMT1 is dependent on the release of the enzyme from its substrates following methylation [67]. These findings suggest a dynamic mechanism for the regulation of substrate methylation that is dependent on the methylation status of its substrates, in this case, BRCA1.

Upon hypomethylation of BRCA1, increased binding to Sp1 protein was observed. The Sp1 transcription factor is a potent transactivator of the insulin-like growth factor-I receptor (IGF-I-R) gene. Initially, the functional interaction between BRCA1 and Sp1 was suggested to regulate the IGF-I-R, a receptor overexpressed in most breast cancers that serves as an antiapoptotic factor [69], [70]. Later, the same group showed that BRCA1 itself does not exhibit any specific binding to the IGF-I-R promoter but instead, it prevented Sp1 binding the promoter by BRCA1-Sp1 interaction at the BRCA1 260–802 region [50]. Moreover, it has been found that BRCA1 gene expression is regulated by the IGF-I signaling pathway where IGF-I enhances BRCA1 promoter activity and that Sp1 is directly involved in BRCA1 gene transactivation [71]. Thus, it is possible that the status of BRCA1 methylation plays a role in the transcriptional regulation of the IGF-I-R gene by BRCA1 and Sp1. Because BRCA1 has an inhibitory control of the IGF-I-R promoter as well as repressing the Sp1-induced transactivation of the IGF-I-R gene, this suggests that methylated BRCA1 state (as observed in our cancer cell lines and breast tumor tissues) allows for decreased BRCA1-Sp1 binding which could be part of a regulatory mechanism of the IGF-I-R gene expression. The *in vivo* interaction of Sp1 with the IGF-I-R promoter in MDA-MB-231 cells treated or untreated with AdOx still needs to be characterized. We have however, observed decreased BRCA1 binding at APEX promoter, which is regulated by Sp1. APEX participates in base excision repair through the recognition and the initial step toward removing abasic sites [72]. The APEX promoter contains Sp1 binding sites both upstream and downstream of the transcriptional start site [73], [74], [75]. Sp1 binding to the downstream site regulates the expression of APEX in a cell cycle dependent fashion [75]. Interestingly, our results indicate that hypomethylated BRCA1 binds to the APEX promoter and that this form of BRCA1 has increased binding to Sp1. Therefore it is possible that BRCA1 is acting as a transcriptional coactivator for Sp1 mediated APEX transcription. The precise role of BRCA1 in APEX transcription will be the focus of future studies.

In our current study we have identified a novel posttranslational modification of BRCA1, namely methylation. We have only begun to elucidate the role of arginine methylation in terms of regulating BRCA1 protein-protein interactions and its transcriptional coactivator function. Future studies will be focused on the identification of the methylated residues, study of lysine methylation, and determining if there is a correlation between BRCA1 methylation and tumor progression. As was the case for BRCA1 phosphorylation, this study is the beginning and multiple future research efforts are needed to uncover the intricate workings of BRCA1 methylation.

## Materials and Methods

### Ethics Statement

The breast tumor samples were previously collected frozen additional extraneous tissue that resulted following testing of breast cancer samples for the presence of hormone receptors. Samples were collected between 1985 and 1995 for pathologic examination and diagnosis. Existing discarded tissue samples were stripped of all identifying information. Ethical approval for the study was given by the George Washington University IRB Committee based on Federal Regulation 45 CFR 46.101(b) (4). The IRB committee review indicated that informed consent was not required as this was additional tissue left over from routine pathological examination.

### *In silico* BRCA1 analysis

Predicted methylation sites from the full length primary amino acid sequence of BRCA1 were generated by the freeware MeMo: Methylation Modification Prediction Server (http://www.bioinfo.tsinghua.edu.cn/~tigerchen/memo.html).

Sequence analysis was performed using the Clustal W multiple sequence alignment program [76]. *Pan troglodytes* (NCBI AAR04849), *Macaca mulatta* (NCBI AAT44833), *Pongo pygmaeus* (NCBI AAT44834), *Gorilla gorilla* (NCBI AAT44835), *Mus musculus* (NCBI AAD00168) and *Rattus norvegicus* (NCBI AAC36493) sequences were compared to human full length BRCA1 (NCBI NP_009225).

### Cell culture and breast tumor tissue samples

MDA-MD-231 and MCF-7 are epithelial breast carcinoma cells derived from pleural effusions [59]. HeLa is a cervical carcinoma cell line commercially available from the ATCC. All cells were grown in Dulbecco's modified Eagle's medium supplemented with 10% FBS, 1% L-glutamine, and 1% streptomycin/penicillin. All cells were incubated at 37°C and 5% CO_2_. Cells were cultured to confluency, washed and pelleted at 4°C for 15 minutes at 3,000 rpm. Pellets were lysed in a buffer containing Tris-HCl pH 7.5, 120 mM NaCl, 5 mM EDTA, 0.5% NP-40, 50 mM NaF, 0.2 mM Na_3_VO_4_, 1 mM DTT and one tablet complete protease inhibitor cocktail per 50 ml. Lysis was performed under ice-cold conditions, incubated on ice for 30 minutes and spun at 4°C for 5 minutes at 14,000 rpm. Supernatant was transferred to a new tube and protein was quantitated with Bradford protein assay (BioRad, Hercules, CA, USA). For the breast tumor tissue lysis, the tissue was ground with a mortar and pestle in the presence of liquid nitrogen to create a powdered tissue. RIPA buffer (50 mM Tris-HCl pH 7.5, 105 mM NaCl, 1% NP-40, 1% sodium deoxycholate, 0.1% SDS, 2 mM EDTA) was added to the powdered tissue, the sample vortexed for 60 seconds, and placed on ice for 45 minutes. Samples were homogenized with a syringe and needle, followed by centrifugation at 14,000 g for 10 minutes. Supernatants containing the lysate were quantitated with Bradford protein assay (BioRad).

### Cell Synchronization

MDA-MB-231 cells were synchronized by treatment with nocodazole (200 ng/ml) for 24 hours. Following treatment, arrested cells were collected by the mitotic shake method. Adherent cells were not collected. One fourth of the cells were collected (M phase population) and the remaining cells were released in Dulbecco's modified Eagle's medium supplemented with 20% FBS, 1% L-glutamine, and 1% streptomycin/penicillin. Cells were collected at 4 hours (G1 phase), 12 hours (S phase), and 20 hours (G2/M phase). Whole cell protein extracts from synchronized MDA-MB-231 cells were separated on a 4–20% gel by SDS-PAGE, and western blotted with antibodies against Cyclin B(H-433), Cyclin D1(M-20), Cyclin E(C-19), cdk4(H-303) and actin(C-11).

### Western blot analysis

Cell extracts were resolved by SDS PAGE on a 4–20% tris-glycine gel (Invitrogen, Carlsbad, CA, USA). Proteins were transferred to polyvinylidene difluoride microporous membranes using the iBlot dry blotting system as described by the manufacturer (Invitrogen). Membranes were blocked with Dulbecco's phosphate-buffered saline (PBS) 0.1% Tween-20+3% BSA. Primary antibody against specified proteins was incubated with the membrane in blocking solution overnight at 4°C. Western blots were performed with a 1∶1 of anti-dimethyl arginine (7E6) (Novus, Littleton, CO, USA) and anti-methyl mono/di arginine (AbCam, Cambridge, MA, USA), anti-methyl lysine (AbCam), anti-PRMT1 (Cell Signaling, Danvers, MA, USA) and anti-BRCA1(C-20), anti-Sp1(16C) and anti-STAT1 (E-23) (Santa Cruz, Santa Cruz, CA) antibodies. Membranes were washed twice with PBS+0.1% Tween-20 and incubated with HRP-conjugated secondary antibody for one hour in blocking solution. Presence of secondary antibody was detected by SuperSignal West Dura Extended Duration Substrate (Pierce, Rockford, IL, USA). Luminescence was visualized on a Kodak 1D image station.

### Immunoprecipitation assay

For immunoprecipitation (IP) 1–4 mg of whole cell protein or tumor tissue extracts were brought up to a final volume of 500 µl with lysis buffer and precleared for 30 minutes with 50 µl of 30% A/G agarose bead slurry (CalBioChem, La Jolla, CA). Supernatants were transferred to a new tube with 10 µg of BRCA1 or normal rabbit IgG antibodies (Santa Cruz), and the solution was rotated overnight at 4°C. The next day complexes were precipitated with A/G beads for 90 minutes. Beads were washed twice with TNE_150_+0.1% NP-40 and once with TNE_50_+0.1% NP-40.

### GST pull-down and in vitro methyltransferase assay

GST tagged proteins were purified as described previously [53]. Constructs were washed three times with PBS+1% Triton X-100, pelleted and resuspended in the methyltransferase buffer reaction. Five hundred nanograms of GST-BRCA1 1–500, 452–1079, 504–802, 697–1276, 1021–1552, 1501–1861 and core histones were incubated with 0.2 µg of recombinant purified PRMT1 (Active Motif, Carlsbad, CA, USA) in the presence of 0.55 µCi S-Adenosyl-L-[*methyl*-^3^H] methionine (GE Healthcare, Piscataway, NJ, USA) and reaction buffer (50 mM Tris-HCl pH 8.0, 20 mM KCl, 10 mM MgCl_2_, 250 mM sucrose, 10 µM β-mercaptoethanol) overnight at 37°C in a final reaction volume of 20 µl. The overnight methylation reactions (beads containing substrate) were spun, washed three times in excess cold 10% TCA, 1% sodium phosphate followed by once with 100% ethanol. Control samples were spotted on GF/C membranes (Millipore, Bedford, MA, USA), allowed to dry and processed equally as beads alone. Both beads and filters were counted in Beckman Coulter LS6001C scintillation counter in 2 ml of scintillation fluid (Beckman Coulter, Fullerton, CA, USA). For the protein GST-BRCA1 pull-down, 2 mg of HeLa whole cell protein extract were brought up to a final volume of 500 µl with lysis buffer and 500 ng of GST-BRCA1 constructs were rotated at 4°C overnight. Beads were washed twice with TNE_150_ + 0.1% NP-40 and once with TNE_50_ + 0.1% NP-40.

### siRNA-mediated knockdown of PRMT1 in HeLa cells

HeLa cells (1.8×10^7^ cells) were transfected with double-stranded duplex with Hs_HRMT1L2_7 HP Validated siRNA (Qiagen, Valencia, CA, USA) or luciferase (Dharmacon, Lafayette, CO, USA) using Lipofectamine reagent according to the manufacturer's recommendations (Invitrogen). Initial transfections were carried out in 24-well plates in order to establish optimal knockdown conditions by titrating siRNA at 10, 25 and 50 nM. Transfections for chromatin immunoprecipitation assays were conducted at 50 nM final siRNA concentration.

### Chromatin immunoprecipitation assay (ChIP)

MDA-MB-231 cells were treated with 30 µM adenosine periodate methyltransferase inhibitor (AdOx, Sigma, St Louis, MO, USA) and processed 48 hours later for ChIP using an established protocol. Approximately 5×10^6^ cells were used per IP. Cells were cross-linked with 1% formaldehyde at 37°C for 10 minutes, pelleted, washed, and cells lysed using SDS lysis buffer (1% SDS, 10 mM EDTA, 50 mM Tris-HCl, pH 8.0, one tablet complete protease inhibitor cocktail per 50 ml) on ice for 10 minutes. Cells were sonicated on ice for 6 cycles to obtain an average DNA length of 500 to 1200 bp. Lysate was clarified by centrifugation at 4°C for 10 minutes at 14,000 rpm. Supernatant was then diluted 10 fold in ChIP dilution buffer (0.01% SDS, 1.1% Triton X-100, 1.2 mM EDTA, 16.7 mM Tris-HCl, pH 8.0, 167 mM NaCl) and pre-cleared with a mixture of protein A/G agarose (blocked previously with 1 mg/ml salmon sperm DNA and 1 mg/ml BSA, Stratagene, La Jolla, CA, USA) at 4°C for 1 hour. Pre-cleared chromatin was incubated with 10 µg of antibody at 4°C overnight. Next day, 60 µl of 30% slurry of blocked protein A/G agarose was added and complexes incubated for 2 hours. Immune complexes were recovered by centrifugation and washed once with low salt buffer (0.1% SDS, 1% Triton X-100, 2 mM EDTA, 20 mM Tris-HCl, pH 8.0, 150 mM NaCl), twice with high salt buffer (0.1% SDS, 1% Triton X-100 2 mM EDTA, 20 mM Tris-HCl, pH 8.0, 500 mM NaCl), once with LiCl buffer (0.25 M LiCl, 1% NP-40, 1% deoxycholate, 1 mM EDTA, 10 mM Tris-HCl, pH 8.0), and once with TE buffer. Immune complexes were eluted twice with elution buffer (1% SDS, 0.1 M NaHCO3) and incubating at room temperature for 15 minutes on a rotating wheel. Cross-links were reversed by adding 20 µl of 5M NaCl and incubating elutes at 65°C overnight. The next day, proteinase K (100 µg/ml final concentration) was added and samples incubated at 55°C for 1 hour. Samples were extracted with phenol:chloroform twice and ethanol precipitated overnight. Pellets were then washed with 70% ethanol, dried, resuspended in 50 µl of TE and assayed by PCR. Thirty-five cycles of PCR were performed in 50 µl with 10 µl of immunoprecipitated material, 0.1 µM of primers, 0.2 mM dNTPs, and 1 unit of Taq DNA polymerase. Finally, PCR products were electrophoresed on 2% agarose gels and visualized by ethidium bromide staining.
